# Supplementary material for: Maternal Genetic Ancestry and Legacy of 10th Century AD Hungarians
Source: Sci Rep. 2016 Sep 16;6:33446. doi: 10.1038/srep33446 (PMC5025779; doi:10.1038/srep33446)
Supplement: Supplementary Figures [file srep33446-s2.pdf]

Supplementary Information

Maternal Genetic Ancestry and Legacy of 10th century AD Hungarians

Aranka Csősz[1#], Anna Szécsényi-Nagy[1#*], Veronika Csákyová[2], Péter Langó[3], Viktória Bódis[1], Kitti Köhler[3], Gyöngyvér Tömöry[1§], Melinda Nagy[4], Balázs Gusztáv Mende[1*]

[1] *Laboratory of Archaeogenetics, Institute of Archaeology, Research Centre for the Humanities, Hungarian Academy of Sciences, Budapest, Hungary*
[2] *Department of Botany and Genetics, Faculty of Natural Sciences, Constantine the Philosopher University in Nitra, Nitra, Slovakia*
[3] *Institute of Archaeology, Research Centre for the Humanities, Hungarian Academy of Sciences, Budapest, Hungary*
[4]*Department of Biology, Faculty of Education, J. Selye University in Komárno, Komárno, Slovakia*

[#]*These authors contributed equally to this study*
[§]*Present address: Hungarian Institute for Forensic Sciences, Budapest, Hungary*

**Corresponding authors:* szecsenyi-nagy.anna@btk.mta.hu, mende.balazs@btk.mta.hu

Supplementary Figures

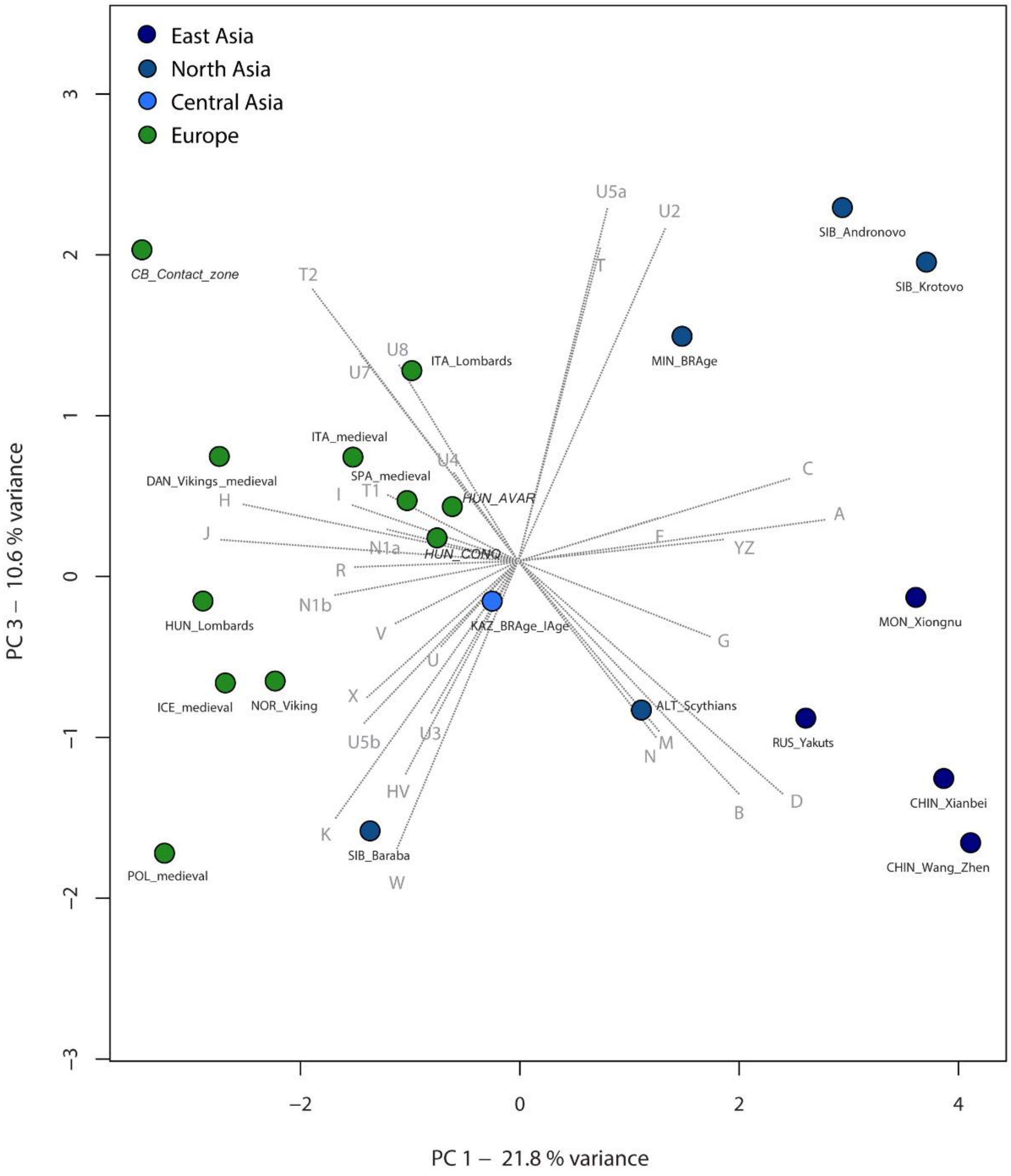

Figure S1. PCA plot of the first and third principal components (32.4% of variance), comparing haplogroup frequencies of 21 ancient populations.

On the PCA plot the contribution of each mtDNA haplogroup is superimposed as grey component loading vector. Along the PC3 (variance = 10.6%), the Avars (HUN_AVAR) and Hungarians from the conquest-period (HUN_CONQ) and the Italian and Spanish (Basque) medieval populations (ITA_medieval; SPA_medieval) remain clustered, while the contact zone sample set (CB_Contact_zone) becomes more adjacent to medieval Europe, than it is along PC2.

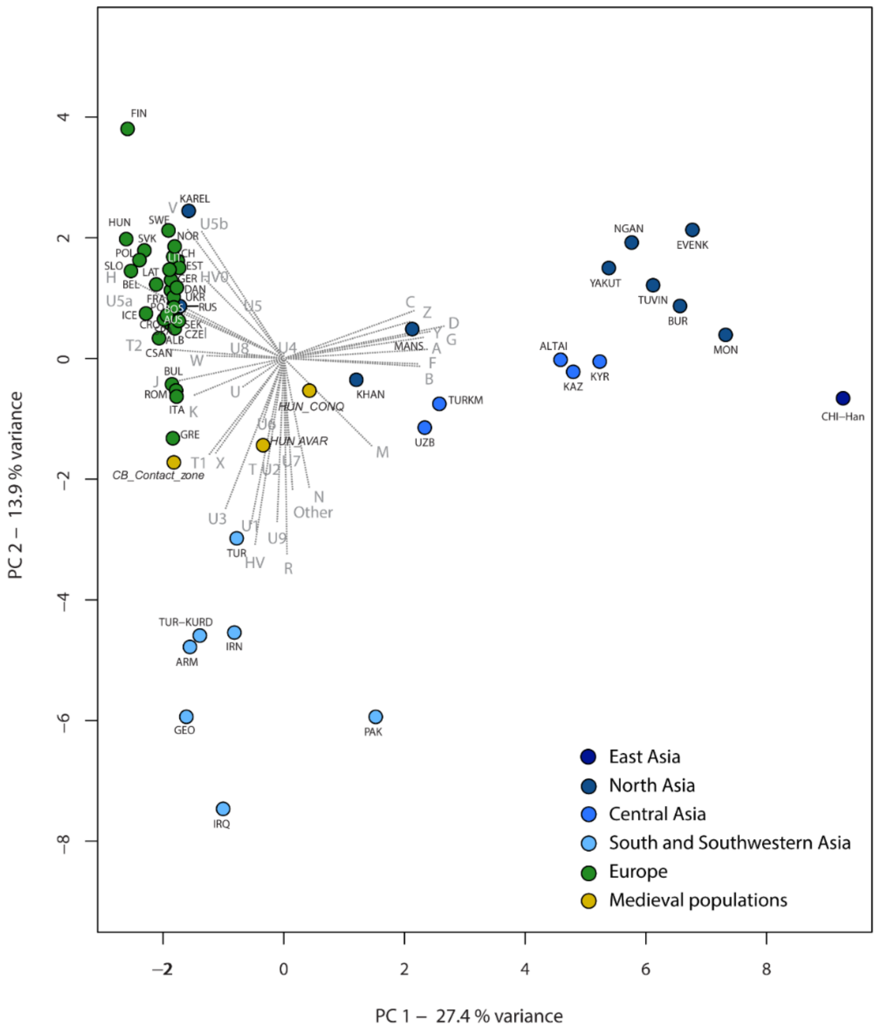

Figure S2. PCA plot with 53 modern populations, representing the first two components (41.3% percent of variance).

The PCA of investigated ancient and modern Eurasian populations displays on the PC1 and PC2 (variance on PC1 = 27.4% and PC2 = 13.9%) the clustering of most European populations in one side, whereas the Asian populations are dispersed along PC1 in the opposite side. Modern-day Near Eastern populations are differentiated along PC2 from Europe. The three investigated medieval population have adjacent positions along PC1-2. Contact zone population (CB_Contact_zone) locates closer to modern-day South Europe, Avars (HUN_AVAR) show affinities toward Turks, and the Hungarian conqueror population (HUN_CONQ) is located in the middle of the plot between Asia and Europe, closest to modern day Turkmenistan, Uzbekistan, and interestingly, to the modern Finno-Ugric populations from Russia (Khanty and Mansi), of which Khanty shows closer connections. For the abbreviations and references see Supplementary Table S6.

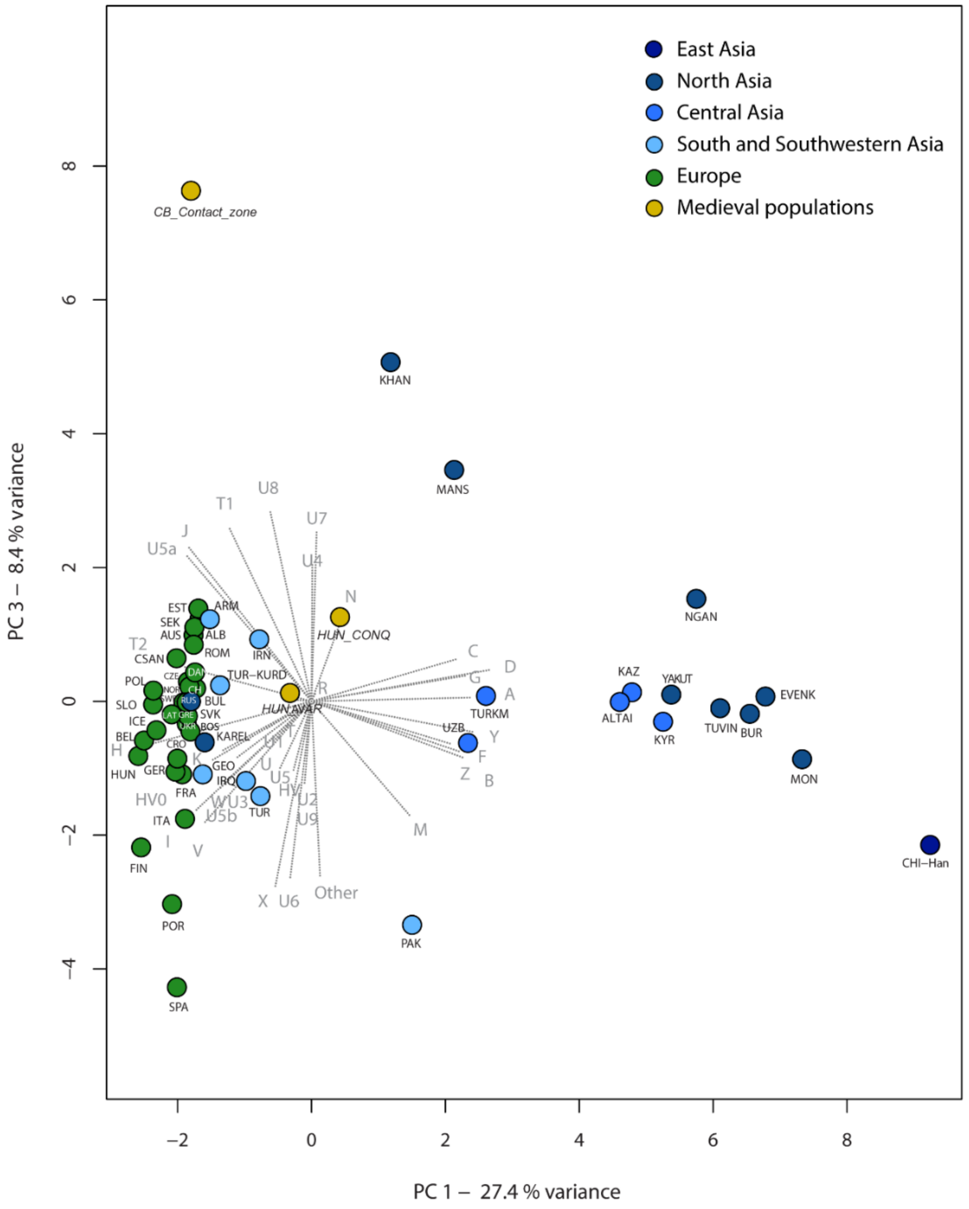

Figure S3. PCA plot with 53 modern populations, representing the first and third components (35.8% percent of variance).

PC3 comprises only low variance proportion (8.4%), but it separates Khanty and Mansi populations from the conquest-period Hungarians (HUN_CONQ), and also the contact zone population (CB_Contact_zone) from Europe. Avars remain close to Europe and to Near East along PC3. Modern day Hungarians show affinities to Poles, Slovenians and Slovaks, their potential Siberian/ Turkic archaic genetic components are not dominant. For the abbreviations and references see Supplementary Table S6.

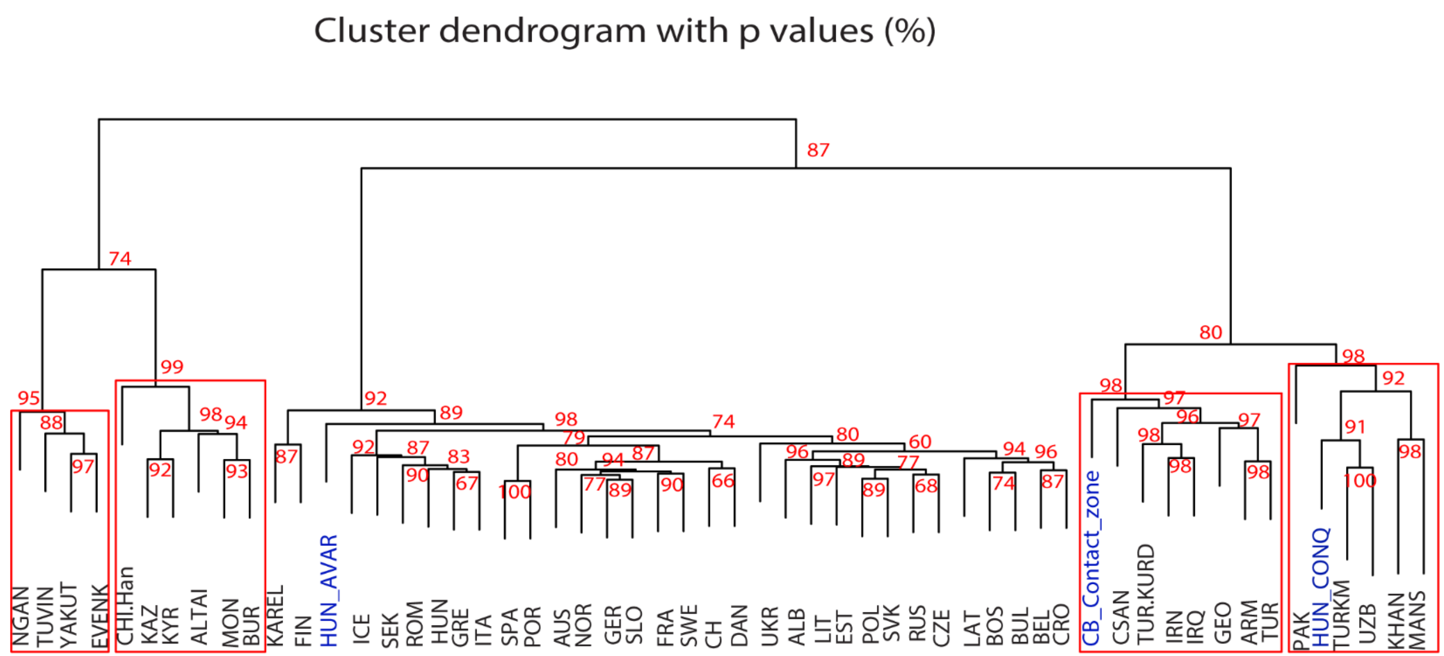

Figure S4. Ward clustering of 53 modern and three medieval populations from the Carpathian Basin.

Ward clustering based on haplogroup frequencies attests the observed relationship of the conquerors on PCAs, but connect contact zone rather to Near East and Avars to Europe. P-values are given in percent as red numbers, red rectangles indicate clusters with significant p values.

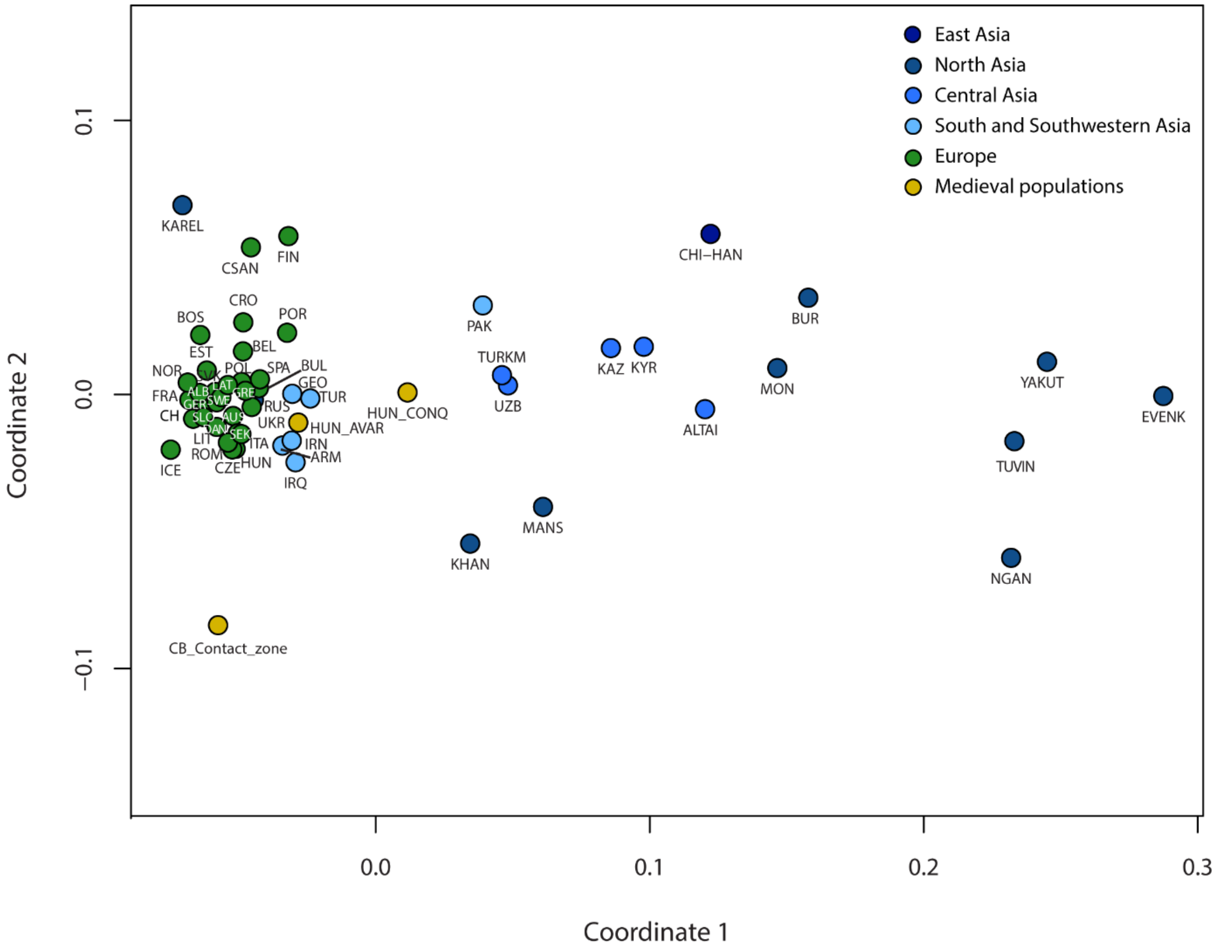

Figure S5. MDS with 52 modern and three medieval populations.

Stress value of the MDS is 0.0640. The Hungarian conquerors (HUN_CONQ) are between Europe/Near East and Central Asia, closest to modern Turks, Turkmens and Uzbeks. The Avars (HUN_AVAR) are more closely to Europeans, within the Near-Eastern pool. The contact zone group (CB_Contact_zone) from the Carpathian Basin is offside, situated toward European populations along coordinate 1, but it is differentiated from most of the populations along coordinate 2. For the abbreviations of modern populations, references and the Slatkin F_{ST} values see Supplementary Table S9.

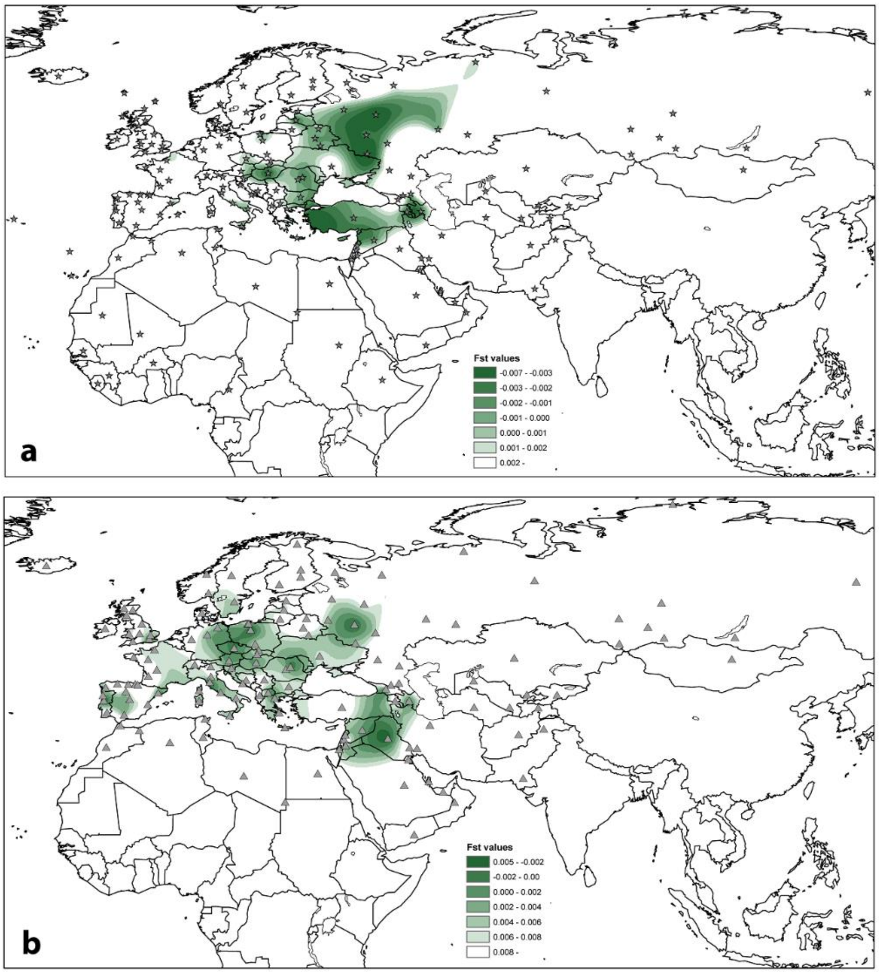

Figure S6. Mapping the genetic distances from the Avars. a: Haplogroup frequency based genetic distances, b: HVS-I sequence based distances.

The genetic distance maps show the European and Near-Eastern or Southwestern Asian, affinity of the Avars. They represent the lowest haplogroup based genetic distances from Easter European populations of Greek Crete, Belgorod, Ivanovo, Yaroslavl and Vladimir Oblast of Russia, Hungary and Latvia, and from Southwestern-Asian populations: Azerbaijan and Armenia (**a**). Furthermore, the Avars show closer affinity to the Kuyavian-Pomeranian population of Poland, Italian Tuscany, Portugal, England, Kaluga, Oryol and Tula Oblast of Russia, Szeklers from Romania, Hungary, Macedonia, Spanish Catalonia and Austria, and to the Near-Eastern and Southwestern Asian populations from Iraq, Georgia, Azerbaijan and to the Turks, in case of haplotype based calculations (**b**). The F_{ST} values and latitude-longitude coordinates were interpolated with Kriging method implemented in Arcmap ArcGIS version 10.3 (https://www.arcgis.com). Grey stars (**a**) and triangles (**b**) signalize the comparative

populations. White regions display F_{ST} values greater than 0.002 (**a**) or 0.008 (**b**) or unconsidered territories. For exact genetic distances see Supplementary Tables S13-14.

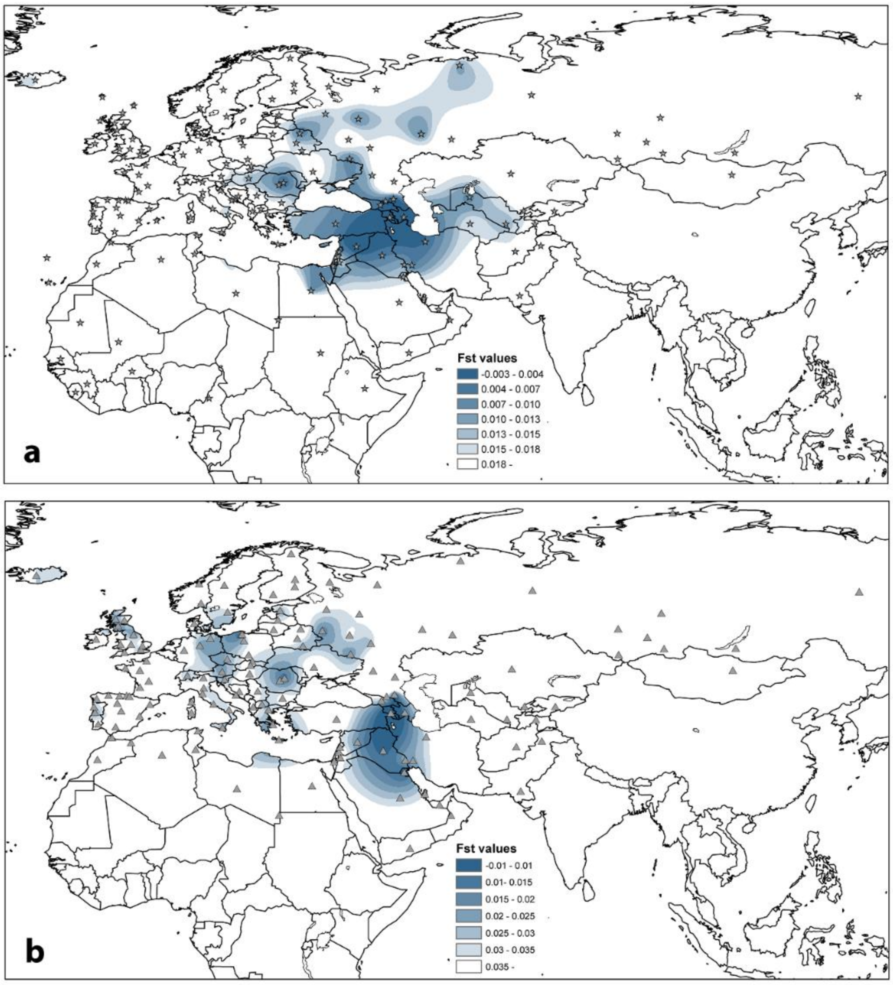

Figure S7. Mapping the genetic distances from the population of the medieval contact zone of the Carpathian Basin. a: Haplogroup frequency based genetic distances, b: HVS-I sequence based distances.

Both genetic distance maps indicate the affinity of the contact zone group to the Southwestern Asia and Near-East: populations, such as of Armenia, Azerbaijan, North Caucasus, Syria, Kuwait and Iraq. Interestingly, the Szeklers are the nearest from Europe to the contact zone. The F_{ST} values and latitude-longitude coordinates were interpolated with Kriging method implemented in Arcmap ArcGIS version 10.3 (https://www.arcgis.com). Grey stars (**a**) and triangles (**b**) signalize the comparative populations. White regions display F_{ST} values greater than 0.018 (**a**) or 0.035 (**b**) or unconsidered territories. F_{ST} values are listed in Supplementary Tables S13- 14.

List of Supplementary Tables

Table S1. Information on all investigated samples.

Table S2. Sequences of the PCR primers.

Table S3. Obtained mtDNA haplogroups and haplotypes.

Table S4. Grouped mtDNA results used for the statistical analyses.

Table S5. Haplogroup frequencies used for PCA with 21 ancient populations and Fisher's test p values.

Table S6. Haplogroup frequencies used for PCA with 53 modern and three ancient populations.

Table S7. Results of the test of population continuity.

Table S8. F_{ST} values, p values and Slatkin F_{ST} matrix of 21 ancient populations.

Table S9. F_{ST} values, p values and Slatkin F_{ST} matrix of 52 modern and 21 ancient populations.

Table S10. Shared haplotype analyses with 21 ancient populations.

Table S11: Haplotype matches of the Asian haplotypes found in the conqueror, Avar and contact zone populations, compared to the reference database.

Table S12. Shared haplotype analyses among the Hungarian conquerors and 23 modern Eurasian populations.

Table S13. Genetic distances of 157 modern populations from three medieval datasets from the Carpathian Basin, calculated from frequencies of 211 mtDNA subhaplogroups.

Table S14. Genetic distances of 141 modern populations from three medieval datasets from the Carpathian Basin, calculated from HVS-I sequences.

Table S15. References to the comparative ancient and modern populations.

Table S16. HVS-I haplotypes of the researchers.
